# Efficient and high-quality absorption enhancement using epsilon-near-zero cylindrical nano-shells constructed by graphene

**DOI:** 10.1038/s41598-024-55194-3

**Published:** 2024-03-21

**Authors:** Shiva Hayati Raad, Mehdi Afshari-Bavil, Dong Liu

**Affiliations:** 1 Key Laboratory of Atmospheric Optics, Anhui Institute of Optics and Fine Mechanics, Hefei Institutes of Physical Science, Chinese Academy of Sciences, Hefei, 230031 China; 2 Department of Electrical and Computer Engineering, Tarbiat Modares University, Tehran, Iran; 3 Advanced Laser Technology Laboratory of Anhui Province, Hefei, 230037 China

**Keywords:** Optics and photonics, Optical materials and structures

## Abstract

This paper presents a detailed scattering analysis of a hollow-core plasmonic-shell cylindrical wire to design an efficient, compact, narrowband, and reconfigurable optical absorber. The shell is formed by a thin graphene material, investigated in its epsilon-near-zero (ENZ) plasmonic region. Compared to the graphene plasmonic resonances in the terahertz(THz)/far-infrared (FIR) frequencies, the ENZ plasmonic resonances offer a blue shift in the operating frequency of the second-order plasmonic resonances by increasing the geometrical dimensions. This feature is successfully used to design efficient optical wave absorbers with absorption cross-sections much larger than geometrical and scattering cross-sections. The observed blue shift in the resonance spectrum, which is the key point of the design, is further verified by defining each particle with its polarizability and fulfilling the resonant scattering condition in the framework of Mie’s theory. Furthermore, graphene relaxation time and chemical potential can be used to manipulate the absorption rate. Observed resonances have narrow widths, achieved with simple geometry. To consider more practical scenarios, the one-dimensional arrangement of the cylindrical elements as a dense and sparse array is also considered and the design key point regarding graphene quality is revealed. The quality factor of the sparse array resonance is 2272.8 and it demands high-quality graphene material in design. It is also observed that due to the use of small particles in the design, the near-field and cooperative effects are not visible in the absorption cross-section of the array and a clear single peak is attained. This polarization-insensitive absorber can tolerate a wide range of incident angles with an absorption rate above 90%.

## Introduction

Localized surface plasmon resonances of noble metals can be excited in cylindrical geometries as a result of the material negative permittivity, provided by the bulk metal (e.g., gold and silver)^1^. These collective oscillations are damped due to the emission of their energy, leading to light scattering, or they are damped non-radiatively as a result of the conversion of the absorbed light into heat. Thus, they offer the feasibility of wave manipulation for a wide range of applications, by changing their size, shape, composition, and environment^2,3^. For instance, scattering cancelation, which is the base of the plasmonic cloaking, can be achieved using semiconductor/metal core/shell nanowires as a result of anti-parallel local polarization vectors in the core and shell^4^. On the other side, scattering enhancement, essential for the super scattering phenomena, is the result of coinciding multipole resonances of the plasmonic wires^5^. It is also demonstrated that anomalous scattering, i.e. the enhanced and suppressed scattering, can be simultaneously observed in multilayer plasmonic cylinders in different wavelengths^6^. Furthermore, non-radiative plasmonic modes in metal-cladded dielectric cylinders with submicron diameters can be used to model coated tips in nearfield scanning optical microscopy^7^. Moreover, chemical and biological sensing applications of plasmonic devices arise from the dependencies of the resonance features on the surrounding environment^8^.

Localized surface plasmon resonance can also be excited in two-dimensional curved graphene sheets, leading to low loss and reconfigurable light-matter interactions^9^. Thus, applications provided by the plasmonic metallic wires can be achieved by replacing the bulk metal with a thin cover^10^. For instance, substantial scattering reduction in the spectral window of the graphene-wrapped cylinders is proposed for tunable invisibility in far infrared frequencies^11^. Also, the electromagnetic wave field absorption enhancement in graphene wires occurs when the linearly polarized incident radiation resonantly excites plasmonic resonances^12^. The refractive index sensing capabilities of the graphene-wrapped wire/tubes are due to the resistive boundary, supporting the localized surface plasmon resonances^13^. Moreover, low loss and highly confined surface wave propagation can be obtained using electrically/magnetically biased graphene shells^14^.

Regarding the operating spectrum, the plasmonic resonances of the noble metals are excited in the visible-near infrared-ultraviolet spectrum, where the losses are the main barrier to their practical usage^15^. The idea of engineering surface plasmons for lower frequencies, is suggested by cutting grooves on the metal surfaces^16^. On the other side, the plasmonic resonances of the graphene-based cylindrical devices are excited in the THz/FIR wavelengths and they offer exotic properties such as low loss, high confinement, and tunability^13,17^. In general, the change in the plasmonic resonance frequency and amplitude of the graphene sheet is employed to suggest several related devices at the THz/FIR spectrum such as refractive index sensors, circular dichroism, polarizers, and modulators^18–22^. Benefiting from graphene plasmons at higher frequencies for emerging and potential applications, such as mid-infrared photodetectors and mid-infrared vibrational spectroscopy is of great interest^23^. Thus, the present research aims at the graphene-based absorber design above the THz/FIR spectrum.

There are two approaches to design high-frequency graphene-based device . In the first method, graphene is modeled by an equivalent dielectric^24^, and efficient graphene-based devices are obtained by coupling it with another resonance such as localized plasmons of metallic nanostructures and Tamm plasmon polaritons (TPPs), to increase the electric field around the graphene^25,26^. For instance, the absorption enhancement of the graphene sheet is achieved using a one-dimensional photonic crystal as the result of photon localization^27^. Also, by coupling the graphene sheet with a metal grating, an 85% enhancement in optical absorption is reported^28^. Moreover, the outer surface of a dielectric-metal core–shell resonator is wrapped with graphene material to enhance its optical absorption cross-section^29^. Alternatively, by wrapping the graphene around a metal-dielectric core–shell nanostructure, unconventional Fano resonance is observed in its absorption response^30^. Critical coupling with a guided mode resonance is another approach for achieving efficient absorption in graphene sheets^31^. Graphene-based metamaterials also lie in this category^32–34^. In the second method, plasmonic resonances of the graphene at NIR–VIS are considered and their influence on the device performance is investigated. Importantly, the evidence for the existence of graphene surface plasmons on a tapered graphene-silicon waveguide tip is studied at a NIR wavelength, employing a surface carrier transfer method, which was impossible to illustrate using current solid back electrical gating and chemical doping methods^35^.

To propose novel graphene-based plasmonic devices far away from the THz/FIR frequencies, it is important to consider the wavelengths in which graphene’s equivalent bulk permittivity remains negative. This gate-tunable region is extended up to around the negative to positive permittivity transition frequency, where the device can also benefit from epsilon near zero constitutive parameter at the end of the interval^36^. The excited resonances are so-called epsilon-near-zero plasmonic resonances and have also been reported for thin conducting oxides such as indium thin oxide (ITO). The excitation of highly confined electromagnetic fields and ultrafast modulation speeds are reported in this region^37–39^. In the vicinity of the ENZ point, unlike the metals, graphene can support TE-like modes with the fractional energy confined to the graphene sheet^40^. Graphene can be an ideal indium-tin oxide substitute due to its enhanced electrical conductivity and high transparency in visible and near-infrared spectra. Solution-processed reduced graphene oxide sheets offer a large-area deposition method for this purpose^41^. Also, reduced graphene oxide micro-meshes are laser-patterned on a plastic substrate and are incorporated to improve and simultaneously tune the optoelectronic properties of graphene-based transparent conductive devices^42^.

The present research aims to use the ENZ plasmonic modes in graphene material to design compact, efficient, tunable, and narrowband optical wire absorbers. The design key point relies on observing the blue shift of the resonance spectrum by increasing the particle size, which makes it feasible to reach low-scattering absorbers due to the use of small particles in the design. This anomalous observation is due to the use of second-order plasmonic resonances in the design and it is also confirmed by employing Mie’s theory. The paper is organized as follows. In "Absorption enhancement using epsilon-near-zero cylindrical shells constructed by graphene" section, the absorption efficiency of a single infinite-length cylindrical particle with an ENZ shell is discussed and the graphene optical parameters selection guidelines are provided. Later, the idea is extended to a dense/sparse array of particles in a one-dimensional arrangement and the impact of the interelement coupling in the absorption rate is revealed. Concluding remarks are mentioned in "Conclusion" section.

## Absorption enhancement using epsilon-near-zero cylindrical shells constructed by graphene

In this section, the absorption efficiency of the cylindrical shells with near-zero negative permittivity, obtained by exploiting graphene material, is studied. The absorption efficiency is calculated regarding the geometrical size and scattering cross-section of the wire and the radius of the wire is selected such that both types of efficiencies are maximized. Then, a one-dimensional array of the investigated elements is considered, and control of the absorption rate regarding the quality of the available graphene material is discussed. The resonance quality factor for different array periodicities is also calculated.

### Absorption/scattering cross-section of an isolated epsilon-near-zero shell

**Figure 1:**
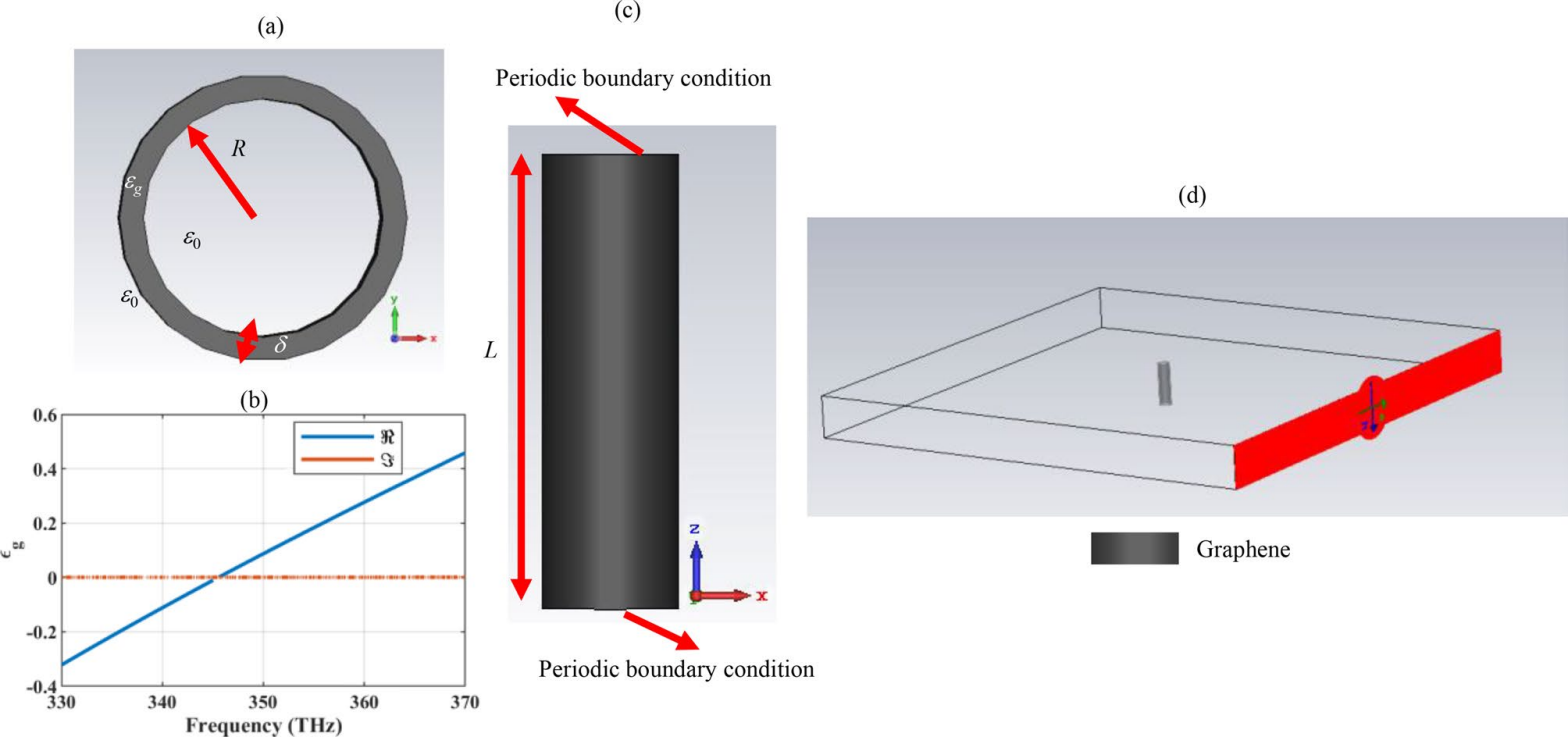
(**a**) The two-dimensional view of the hollow core cylindrical wire with the radius *R*, wrapped by the graphene material with the thickness *δ* = 1 nm. (**b**) The real $\left( \Re \right)$ and imaginary $\left( \Im \right)$ parts of graphene equivalent bulk permittivity *ε*_*g*_ in the desired frequency range, for illustrating its epsilon near zero region. (**c**) The three-dimensional view of the finite-length graphene-coated wire, simulated by applying the periodic boundary condition along its axis to resemble an infinite-length cylinder, and (**d**) illustrating the incoming plane wave polarization for the possibility of plasmonic excitation.

Let us consider a hollow core cylindrical wire with the radius *R,* coated with a graphene shell with the thickness *δ* = 1 nm, as shown in Fig. 1a. The selected thickness is based on the reported experimental data for the graphene sheets grown by chemical vapor deposition (CVD)^26^. Note that there are several methods for graphene production including: from graphite through mechanical and liquid phase exfoliation, chemical vapor deposition, solvothermal synthesis from organic compounds, chemical cross-linking of polycyclic aromatic hydrocarbons, thermal decomposition of SiC and carbon nanotube unzipping. The CVD is the most widely used method and it results in less defective films^41^. The extension of the analysis for the graphene shells with other thicknesses is straightforward. Note that graphene can be wrapped around cylindrical particles using the tape-assist transfer method, spin-coating method, and template-assisted method due to the van der Waals forces^43–45^.

To obtain the scattering and absorption cross sections, the device is simulated in the CST software package and it is analyzed by the frequency domain solver. For the sake of simulation, graphene is modeled by its equivalent dispersive bulk permittivity *ε*_*g*_, achieved using the associated surface conductivity *σ*. The surface conductivity is calculated by the Kubo formulas as the summation of intra-band and inter-band contributions as follows^46^:

1 $$\sigma_{{{\text{intra}}}} = \frac{{i2e^{2} k_{B} T}}{{\pi \hbar^{2} \left( {\omega + i\Gamma } \right)}}\ln \left[ {2\cosh \left( {\frac{{\mu_{c} }}{{2k_{B} T}}} \right)} \right]$$

2 $$\sigma_{{\text{inter}}} \approx \frac{{e^{2} }}{4\hbar }\left[ {\frac{1}{2} + \frac{1}{\pi }\arctan \left( {\frac{{E^{ + } }}{{2k_{B} T}}} \right)} \right. - \left. {\frac{i}{2\pi }\ln \frac{{\left( {E^{ + } } \right)^{2} }}{{\left( {E^{ - } } \right)^{2} + \left( {2k_{B} T} \right)^{2} }}} \right]$$

where $E^{ \pm } = \hbar \omega \pm 2\mu_{c}$ and $\hbar$ is the reduced Planck’s constant, $\mu_{c}$ is the chemical potential of graphene, and *e* is the electron charge. Moreover, *k*_*B*_ is Boltzmann’s constant, *T* is the temperature, Γ is the charge carrier scattering rate. If the graphene layer has a very small thickness in comparison to the excitation wavelength, its equivalent bulk permittivity can be calculated via Amper’s law as $\varepsilon_{g} = {{i\sigma } \mathord{\left/ {\vphantom {{i\sigma } {\varepsilon_{0} }}} \right. \kern-0pt} {\varepsilon_{0} }}\omega \,\delta$, where *ε*_0_ is the free-space permittivity and *ω* is the angular frequency. Note that by using the graphene material in the optical design, the layer thickness can be shrunk down to the multiples of carbon atom diameter (0.34 nm). The choice of the 1 nm thickness is inspired by the seminal research in this field, where it is indicated that as long as the considered thickness is extremely small in comparison to the excitation wavelength, its value is not essential. On the other hand, the same results can be obtained either with the 1 nm thick layer or an extremely fine one^47^. By using larger thicknesses in the numerical simulation, the high contrast between geometrical dimensions is avoided. Furthermore, modeling graphene with a 1 nm thick layer with the equivalent permittivity obtained through the Kubo formulas and Amperes’ law can also be found in^36,48,49^. Also, the graphene relaxation time is set to *τ* = 2 ps, and its chemical potential is considered equal to *μ*_c_ = 1 eV in the initial simulations and their influence on the optical response is investigated later. These optical parameters control the quality of the fabricated material and the applied gate voltage, respectively. The provided physical concepts are supported for any value of these parameters. The equivalent permittivity of the graphene material in the frequency range of interest is provided in Fig. 1b, to confirm that it behaves as a very low-loss material, capable of supporting epsilon-near-zero negative and positive permittivities^36^. Also, note that the hollow-core wire consideration does not pose any limitation on the practicality of the device and it ensures that no other resonances, apart from those of the graphene shells, are excited^45,50^. Because, light absorption enhancement is also possible using Whispering gallery modes in dielectric particles^51^. Note that enhanced resonance modes and mobility are also realizable using nanospheres and nanotubes, as well. Graphene sheets can be wrapped around curved surfaces with different shapes and similar functionalities can be achieved with other configurations^52–54^.

Since the cylindrical wire is infinite in length, to use the three-dimensional CST simulating software to analyze a two-dimensional geometry, the cylinder length is set to an arbitrary finite value *L* = 40 nm, and periodic boundary conditions are applied along the cylinder axis (Fig. 1c). In the case of infinite-length cylinders, the scattering cross-section is defined as “the ratio of the scattered power per unit axial length to the incident power density.” Thus, the scattering (absorption) cross-section has the dimension of length^55^. Thus, by normalizing the final results to *L*, the scattering and absorption cross-sections of the infinite-length wire will be achieved. For more details about the simulation setting the reader is referred to^56^. Also, the device is simulated under plane wave illumination and its absorption cross-section (ACS) and scattering cross-section (SCS) are obtained by using far-field monitors. Note that the electric field of the source resides in the cylinder cross-section (Fig. 1d). The proper choice of plane wave polarization is important when employing cylindrical particles with monolithic plasmonic covers in the design. Otherwise, the localized surface plasmon resonances are not excited. To achieve dual polarized functionality, polarization-independent coatings are proposed^57^.

**Figure 2:**
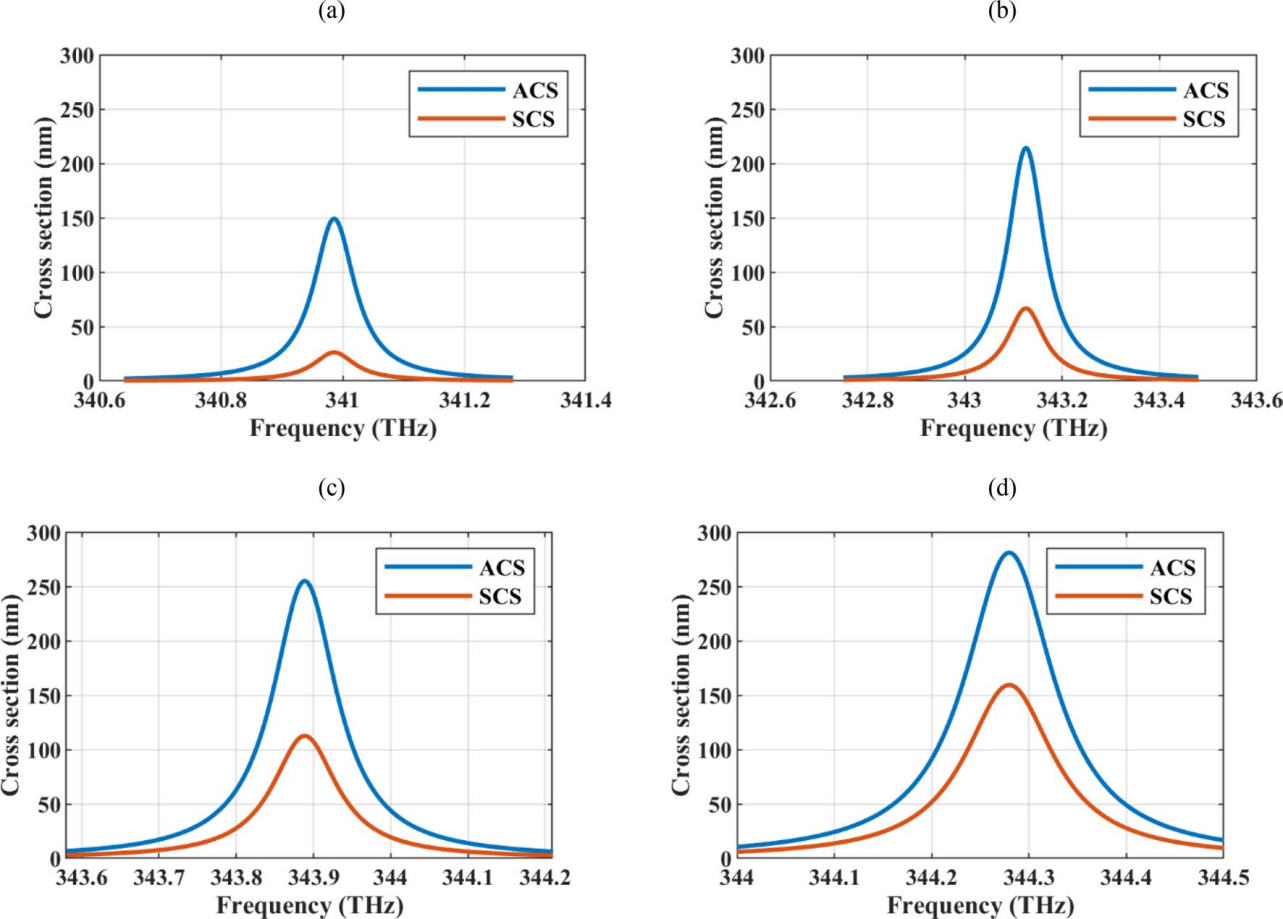
The absorption cross-section (ACS) and scattering cross-section (SCS) of the hollow core graphene-coated wire for different core radii *R* (**a**) 5 nm (**b**) 10 nm (**c**) 15 nm, and (**d**) 20 nm. The graphene sheet thickness is set to δ = 1 nm and its optical parameters are τ = 2 ps and μ_c_ = 1 eV.

Once the quality and bias voltage of the graphene shell is specified, the only degree of freedom for absorption/scattering manipulation is the cylinder radius. Figure 2a–d shows the device's absorption and scattering cross-sections for various core radii. The observed resonances are of the Mie type and can be analyzed in the framework of Mie’s theory^36^. Generally, for the low-index pure dielectric particles of a small size, Rayleigh scattering is observed. Here, these resonances are avoided by considering a hollow core structure. The appearance of the Mie resonances is due to the plasmonic nature of the graphene shell^50^. The results show that the absorption peak blue shifts once the wire radius is increased. Specifically, the resonance frequency for the core radii of *R* = 5, 10, 15, 20 nm are respectively 340.99, 343.13, 343.89, 344.28 THz. This feature is unlike the behavior of graphene-coated wire's plasmonic resonances in the infrared region, so-called ordinary plasmonic resonances^58^. This is the key point for increasing absorption efficiency by reaching a low-scattering device and is observed as a result of using higher-order resonances for the absorber design^59^. To evaluate the performance of the different-sized particles, note that larger particles provide more absorption area. The higher absorption cross-section cannot always be translated into a higher absorption efficiency since larger particles also provide more scattering areas. Thus, two types of efficiency are defined in this regard. The first efficiency is defined as the ratio of the absorption cross-section to the wire circumference 2πR, which gives the scattering per unit length in terms of the cylinder radius^60^. The second type of efficiency takes the scattering cross-section as the normalization constant of the absorption cross-section^61^.

Table 1 shows the numerical values of the defined efficiencies for different core radii and it confirms that the smaller particles have better performance regarding both types of efficiencies. Thus, the wire radius is set to *R* = 5 nm. The electrical radius of the particle is 0.006 λ for this specific radius, confirming the deep-subwavelength operation. Observing strong resonances with small-sized particles is crucial for low-scattering and compact device design. Note that the minimum considered radius is limited to 5 nm to be in the validity region of graphene surface conductivity approximation which is used in the simulations^58^.

**Table 1:** Absorption efficiency of the hollow graphene-coated particles for different radii *R* (*δ* = 1 nm, τ = 2 ps, and μ_c_ = 1 eV).

*R* (nm)	5	10	15	20
ACS (nm)/(2πR)	4.74	3.41	2.70	2.23
ACS (nm)/SCS (nm)	5.71	3.21	2.27	1.76

To further investigate the reason for resonance blue shift by increasing the particle size, the resonant scattering condition for dielectric core metal shell particles is used. By defining the filling fraction as $f = \left( {{R \mathord{\left/ {\vphantom {R {(R + 1)}}} \right. \kern-0pt} {(R + 1)}}} \right)^{3}$, the polarizability of the hollow core particles can be defined as^62^:

3 $$\alpha = 4\pi \frac{{\left( {1 - \varepsilon_{g} } \right)\left( {1 + \varepsilon_{g} } \right)\left( {f - 1} \right)}}{{ - \left( {1 - \varepsilon_{g} } \right)^{2} f + \left( {1 + \varepsilon_{g} } \right)^{2} }}$$

**Figure 3:**
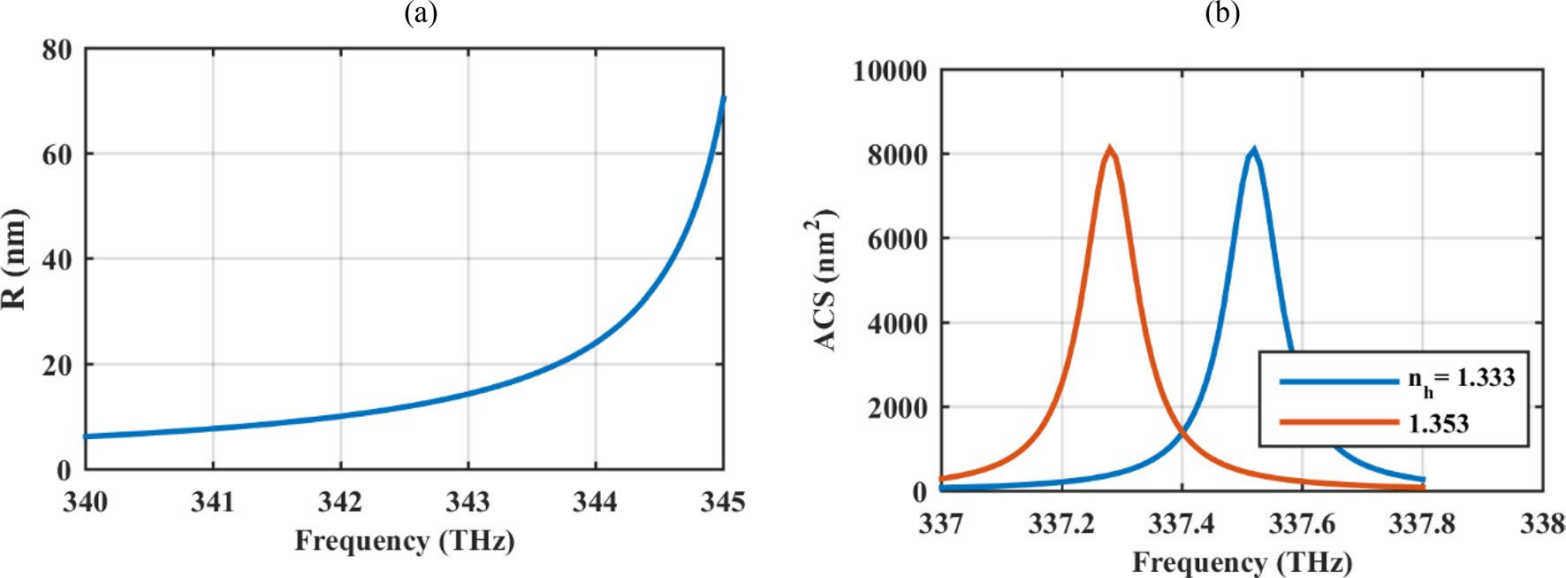
Resonant scattering condition to determine the core radius to observe a resonance in a specific frequency for graphene-coated hollow particles. The graphene sheet thickness is set to *δ* = 1 nm and its optical parameters are τ = 2 ps and μ_c_ = 1 eV. Note that the ACS values are unnormalized.

Since the scattering cross section is proportional to $\left| \alpha \right|^{2}$, to observe resonant scattering, the denominator of the polarizability should approach zero. Figure 3 shows the resonance condition for cores with different radii and it is evident that to observe resonance at higher frequencies, larger particles are required.

In the following, the potential applications of the proposed device will be discussed. Due to the operation of the device in the near-infrared regime, low scattering of excitation and emission light, low levels of autofluorescence in most biological systems and less disturbance and photodamage caused to cells and living organisms is expected in biological applications^63^.The graphene oxide wrapped gold nanoparticles with the approximate radius of 100 nm has been used for drug delivery^64^. Importantly, nanoparticles with hydrodynamic diameters less than 10 nm can be delivered to tumors with higher delivery efficiency compared to larger nanoparticles. Also, nanoparticles smaller than 20 nm in diameter have higher tumor tissue penetration and they are demanded for uniform distribution and reduced self-diffusion barriers because of their small size^65^. Moreover, as another potential application of the absorber, its refractive index sensing capability is investigated by changing the permittivity of the host medium to 1.77689 (*n*_*h*_ = 1.333) and 1.83061 (*n*_*h*_ = 1.3530). The considered refractive index values can be used to model some types of cancer cells in the blood serum^66^. Note that the average refractive index of some normal cells in the near infrared regime are; cytoplasm (1.360–1.375), melanin (1.6–1.7), extracellular fluid (1.35–1.36), nucleus (1.38–1.41). In general, the refractive index of the cancer cells is higher than those of the normal cells due to more protein in their cytoplasm^67^. Thus, depending on the target cell, the refractive index variation range of the biosensor can be chosen and, apart from simulated values, other ranges are also considered in the literature in this regard (e.g., 1.392–1.401 in^68^ and 1.41–1.58 in^69^).

The sensitivity *S* = Δ*λ*/Δ*n* and figure of merit FOM = *S*/FWHM (full width at half maximum) of the sensor are respectively 31.62 nm/RIU and 109.16 RIU^−1^. Δ*λ* and Δ*n* are respectively the change in the resonance wavelength and refractive index. In comparison to the graphene-coated cylindrical particles in ordinary plasmonic regime with *R* = 50 000 nm, *S* = 410 nm/RIU, and FOM = 0.874 RIU^−1^ reported in^13^, our proposed element is very compact, has smaller sensitivity but larger figure of merit. The compact size of the particle with ENZ shell guarantees the low scattering and the narrow beamwidth (due to the use of low loss material) results in a high figure of merit. Note that in any case, higher sensing parameters can be attained by optimizing the performance and the reported values are for the sake of comparing the range of the parameters. In such an optimization, the particle size plays a crucial role in its performance. The nanoparticle size ranges from 10 to 100 nm, which is smaller than the size of the blood cells and almost the same size of DNA^70^. DNA biosensor, designed with the gold nanoparticles with the radius of around 30 nm, is proposed in this regard^71^. Moreover, the size of the particle can be controlled to reach a similar size to that of the biomolecule that it interacts (protein, 5–50 nm; virus, 20–450 nm; cell, 10–100 μm)^72^.

**Figure 4:**
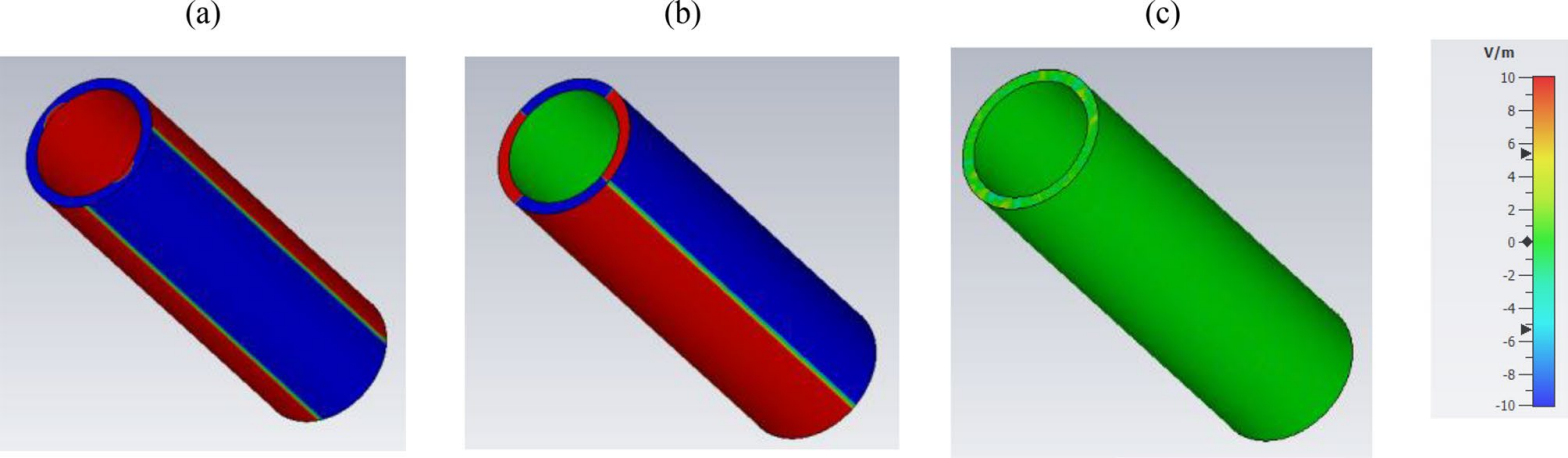
The electric field distribution for the hollow graphene-wrapped optical wire with R = 5 nm, δ = 1 nm, τ = 2 ps, and μ_c_ = 1 eV at the resonance (**a**) E_x_ (**b**) E_y_ and (**c**) E_z_.

To better recognize the operation mechanism, the electric field distribution at the absorption/scattering peak for the hollow wire with *R* = 5 nm, *δ* = 1 nm, *τ* = 2 ps, and *μ*_c_ = 1 eV is shown in Fig. 4a-c. The figure confirms the excitation of the plasmonic resonances at the vicinity of the zero-crossing point of the graphene equivalent bulk permittivity, previously denoted as ENZ plasmonic resonances. The real and imaginary parts of graphene equivalent permittivity at the resonance frequency are respectively − 0.009 and 0.0007. The high confinement of optical plasmonic modes to the graphene in the ENZ region ensures highlight–matter interaction^28^. Apart from charge oscillation, observed in the electric field distributions, the negative signed real part of the near-zero permittivity is another evidence of the plasmonic resonances. Note that the imaginary part of the equivalent permittivity is also near zero, and confirms the low loss performance of the designed structure. It is important to emphasize that due to the low-loss plasmonic behavior of the device, the operating bandwidth is narrow (see Fig. 2). This narrow-band operation is similar to those of the graphene-coated spherical particle’s Fano-like scattering resonance in the epsilon-near-zero spectrum^36^.

Tables 2 and 3 are dedicated to studying the absorption manipulation using graphene optical parameters for the wire with *R* = 5 nm and *δ* = 1 nm. The reported parameters are ACS, SCS, and their ratio. Due to the constant radius in the simulations, the normalization of the ACS to the geometrical cross-section is not reported. In Table 2, the graphene relaxation time is varied in the experimentally achievable range from τ= 0.5–2 ps^73^. Graphene relaxation time is inversely proportional to its scattering rate which is related to the purity of the sheet and can be controlled by experimental method to produce it^36^. As the table shows, the high-quality graphene material (high relaxation time in the realizable range) results in a larger absorption cross-section, but the scattering cross-section is also larger under this condition and may reduce the absorption efficiency. Considering the ACS/SCS values, it is observed that the absorption efficiency is higher for lower graphene relaxation times.

**Table 2:** ACS, SCS, and absorption efficiency of the hollow graphene-wrapped wire with *R* = 5 nm and *δ* = 1 nm for *μ*_c_ = 1 eV.

*τ* (ps)	0.5	1	1.5	2
ACS (nm)	49.03	89.01	121.81	149.03
SCS (nm)	2.22	8.05	16.32	26.25
ACS/SCS	22.04	11.06	7.46	5.67

**Table 3:** ACS, SCS, and absorption efficiency of the hollow graphene-wrapped wire with *R* = 5 nm and *δ* = 1 nm for *τ* = 2 ps.

*μ*_*c*_ (eV)	1	1.02	1.03	1.04
ACS (nm)	148.99	154.74	157.17	159.08
SCS (nm)	26.10	29.35	30.96	32.65
ACS/SCS	5.71	5.27	5.07	4.87
Resonance frequency (THz)	340.99	346.74	349.6	352.45

Regarding Table 3, the chemical potential of the graphene shells is varied around the initially considered value of μ_c_ = 1 eV. Larger absorption and scattering cross-sections can be achieved for higher chemical potentials. Further study reveals that the absorption efficiency is almost the same for different chemical potentials and the chemical potential of the graphene shell can be used to reach a reconfigurable device. The resonance frequency for different values of the chemical potential is also reported in the last row of Table 3. Note that to maintain the same setting for the frequency domain solver, in all simulations, the chemical potential is varied in a small range to observe the plasmonic resonances in close frequencies. Furthermore, typically measured mobilities of processed graphene are as low as 1000 cm^2^/Vs. By encapsulating graphene with molybdenum or tungsten disulfides and hBN, high carrier mobilities of about 60,000 cm^2^/Vs can be achieved. The mobility also depends on the chemical potential and the chemical potentials less than 2 eV are realizable^73,74^. The damping constant $\Gamma_{c}$, which qualifies the losses in the graphene, is related to the mobility $\mu$ and chemical potential $\mu_{c}$ via $\Gamma_{c} = {{q\hbar v_{f}^{2} } \mathord{\left/ {\vphantom {{q\hbar v_{f}^{2} } {\mu \mu_{c} }}} \right. \kern-0pt} {\mu \mu_{c} }}$. The parameters *q*, $\hbar$, and *v*_*f*_ are respectively electron charge, reduced Planck’s constant, and Fermi velocity^36^.

**Figure 5:**
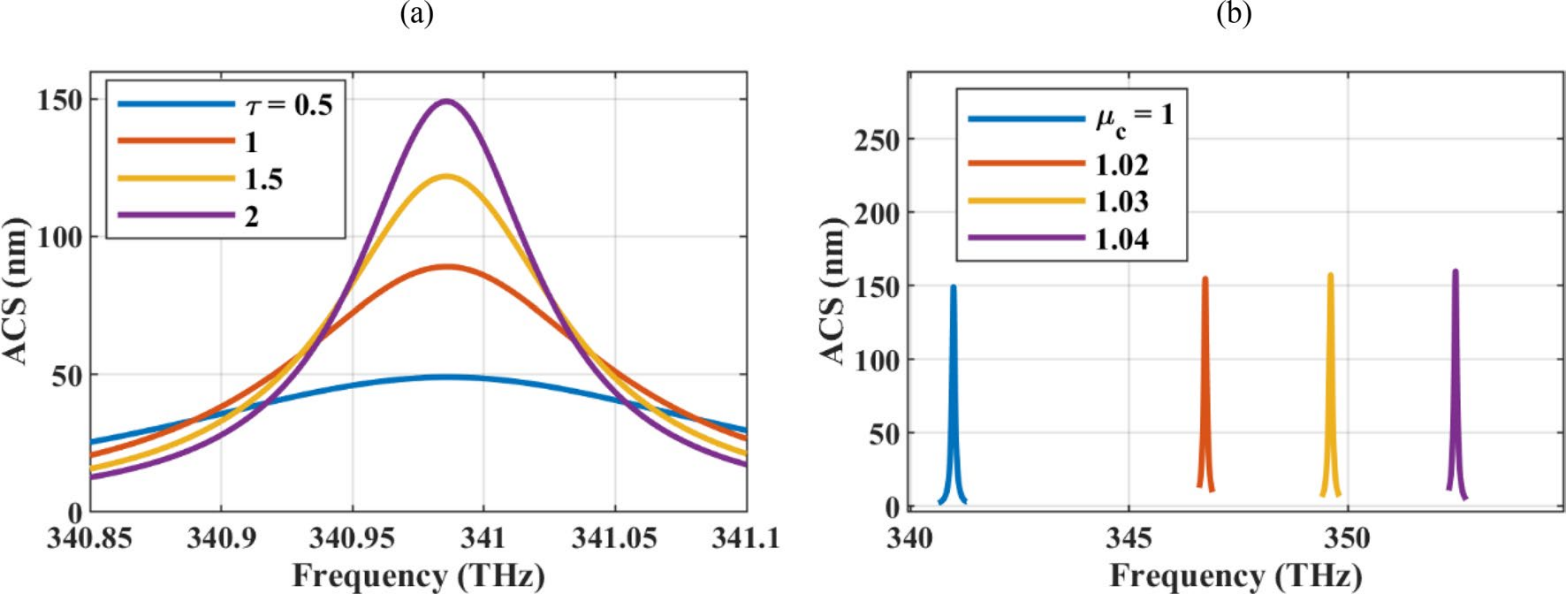
The ACS of the particle for different values of the (**a**) relaxation times and (**b**) chemical potentials. The initial values of the design parameters are R = 5 nm, δ = 1 nm, τ = 2 ps, and μ_c_ = 1 eV and in each sub-figure, one of the parameters is altered.

The ACS of the particle for different values of the relaxation times and chemical potentials are illustrated in Fig. 5a,b to exhibit the impact of the graphene optical parameters in the linewidth modulation.

### Absorption rate of an array of epsilon-near-zero shells

**Figure 6:**
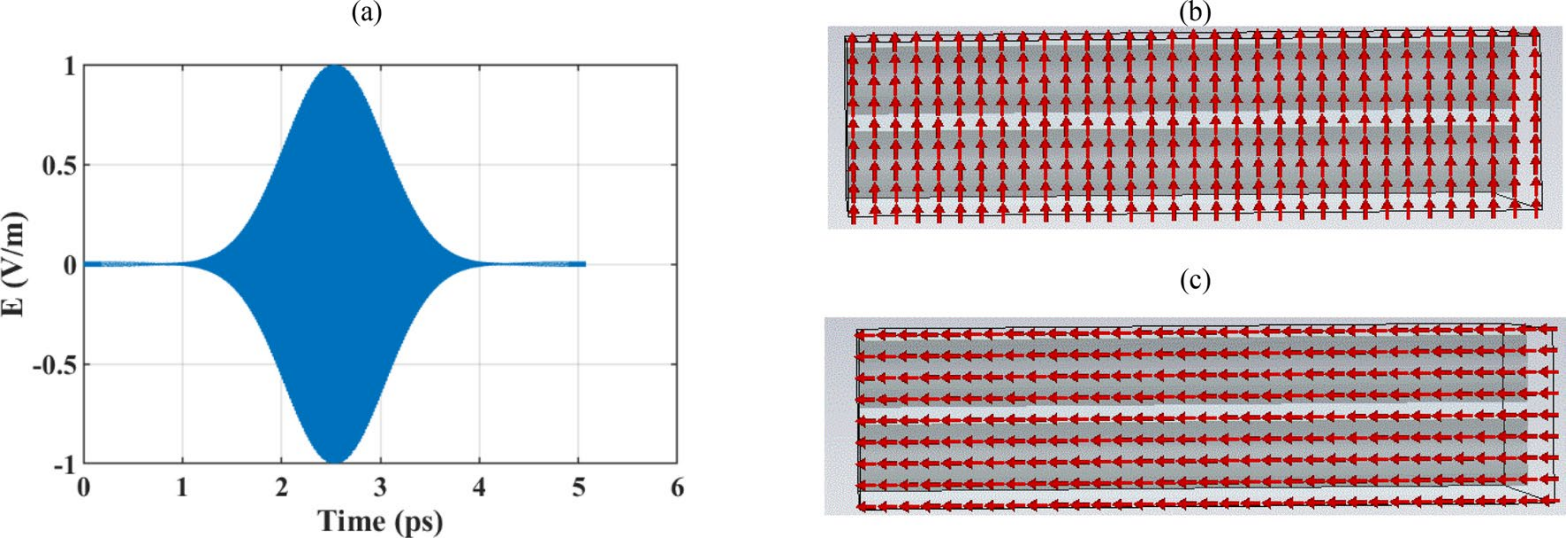
(**a**) The spatial electric field distribution of the Gaussian beam. (**b**–**c**) are respectively the port modes for TE (0,0) and TM(0,0) excitation sources. Only two of the array elements are illustrated.

In the previous section, the efficient tunable absorption of the optical wave using isolated hollow core graphene-coated cylindrical wires, operating based on the epsilon-near zero plasmonic resonances, is revealed. The localized surface plasmon-based optical absorbers are commonly obtained by exploiting an array of coated particles rather than using a single element^75,76^. Structured ultrathin optical materials at the subwavelength scale, provide the opportunity for the reflection and transmission manipulation for the realization of effective light-trapping devices^77^. The high absorption rate is the result of different resonances and mechanisms including but not limited to Mie’s resonances, waveguide resonances, enhanced near field, and total internal reflections from the substrate^77,78^. Hence, considering the mutual interaction of the elements in the plasmonic array is essential for the practical absorber design. Thus, in this section, a one-dimensional array of cylindrical nano-wires with epsilon-near-zero shells is considered and it is shown in Fig. 6a. The absorption is calculated using the scattering parameters by simulation the unit cell of the array under Floquet port excitation^76^. In a periodic structure, the electromagnetic field can be decomposed into a series of Floquet modes, each of which can be thought of as a plane wave propagating at a different angle^79^. Floquet ports enable the simulation of periodic structures more efficiently by allowing to injection or extraction of specific modes or wave components at the boundaries of the periodic structures. Here, the first TE and TM modes are considered as the excitation. The array is illuminated with the Gaussian beams from the top and bottom of the array. The spatial electric field distribution of the gaussian beam is illustrated in Fig. 6a and the electric field of the TE (0,0) and TM (0,0) modes are illustrated respectively in Fig. 6b,c. Note that unit cell analysis is performed in the frequency domain in the CST software.

**Figure 7:**
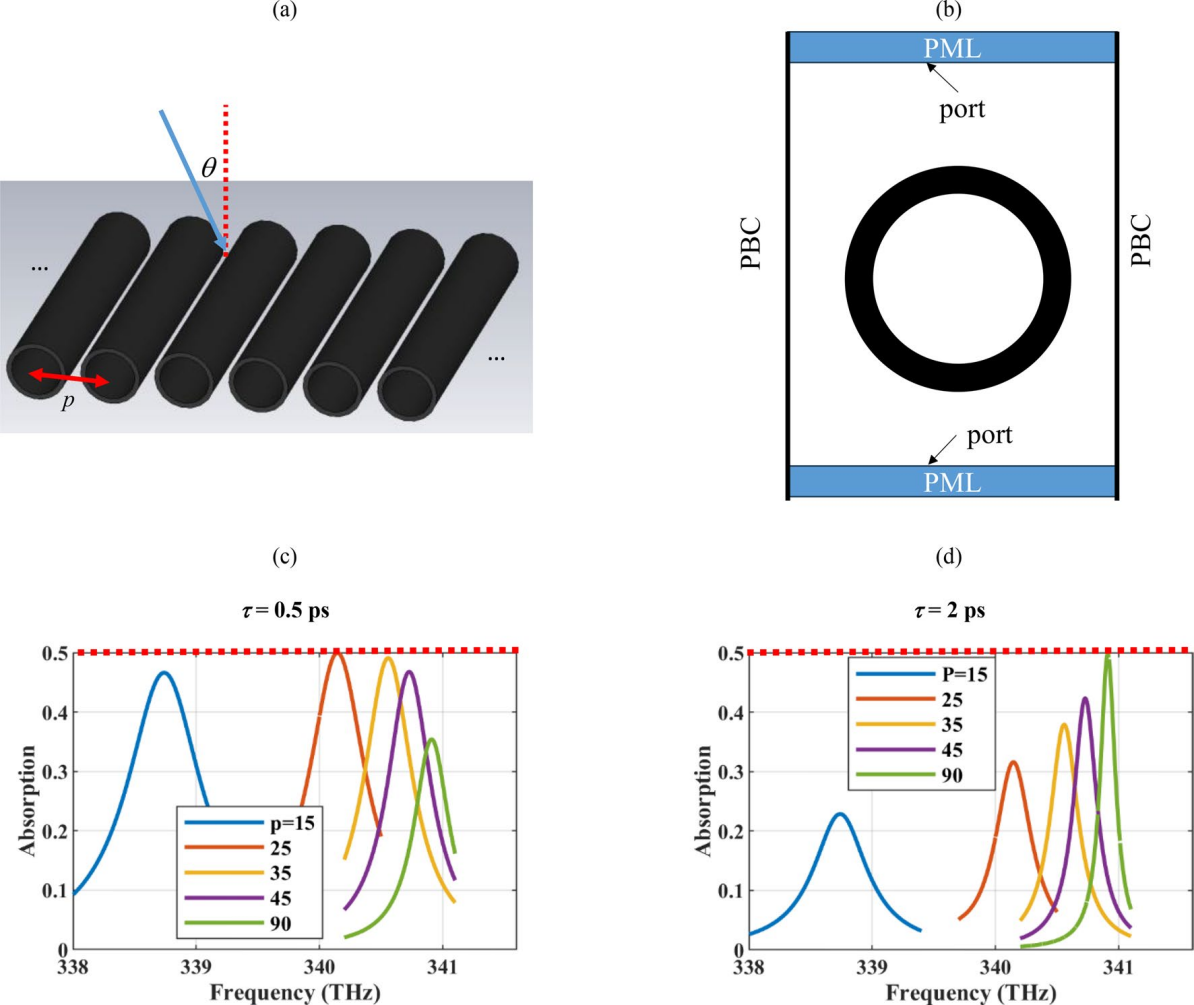
(**a**) One-dimensional array of graphene-coated hollow wires and (**b**) its two-dimensional view. The attainable absorption rate for different periodicity *p* (nm) for (**c**) low-quality (*τ* = 0.5 ps) and (**c**) high-quality (*τ* = 2 ps) graphene covers. The other parameters are *R* = 5 nm, δ = 1 nm, μ_c_ = 1 eV. The maximum attainable absorption rate using free-standing polarizable particles is shown by the dotted red curve in the figure. The PML and PBC respectively denote the perfectly matched layer and period boundary condition.

The array geometry and associated boundary conditions are shown in Fig. 7a,b. The array periodicity *p* is swept in Fig. 7c,d, where the relaxation times of the graphene shells are respectively 0.5 ps (low-quality sheet) and 2 ps (high-quality sheet). The minimum gap distance is set to 5 nm, which is sufficient for neglecting the nonlocal effects, dominant for the gap distances below 2 nm^80^. As the figure confirms, the mutual coupling of the plasmonic resonances for smaller interelement spacings results in the red shift of the resonance spectrum and its broadening^81^. The maximum achievable absorption rate of 50% for an array of free-standing polarizable particles is observed for different optimum periodicities when the quality of the graphene material is altered. This is the universal absorption limit for the thin layers, which can be verified by means of lattice sum and particle polarizability. Full absorption can be attained by supporting the particles on a substrate^82^. Thus, the periodicity of the elements can be adjusted based on the quality of the available graphene material to reach the universal absorption limit. For the dense array with the *p* = 25 nm, the resonance frequency is *f* = 340.14 THz, full-width at half maximum (FWHM) is 1.43 nm, thus the quality factor is *Q* = λ/FWHM = 618.45. For the sparse array (with the resonance frequency very close to that of the isolated wire) the resonance frequency is *f* = 340.91 THz, FWHM = 0.39 nm, hence *Q* = 2272.8. Thus, the achievable resonance quality factor using the sparse arrays is larger and they require high-quality graphene materials. In comparison to the same geometry in the THz spectrum (graphene-coated cylinder array) with FWHM = 86.08 nm^83^, the resonance width had been considerably reduced in both cases. By exploiting other materials such as phase change materials, other functionalities can also be added to the device^84,85^.

**Figure 8:**
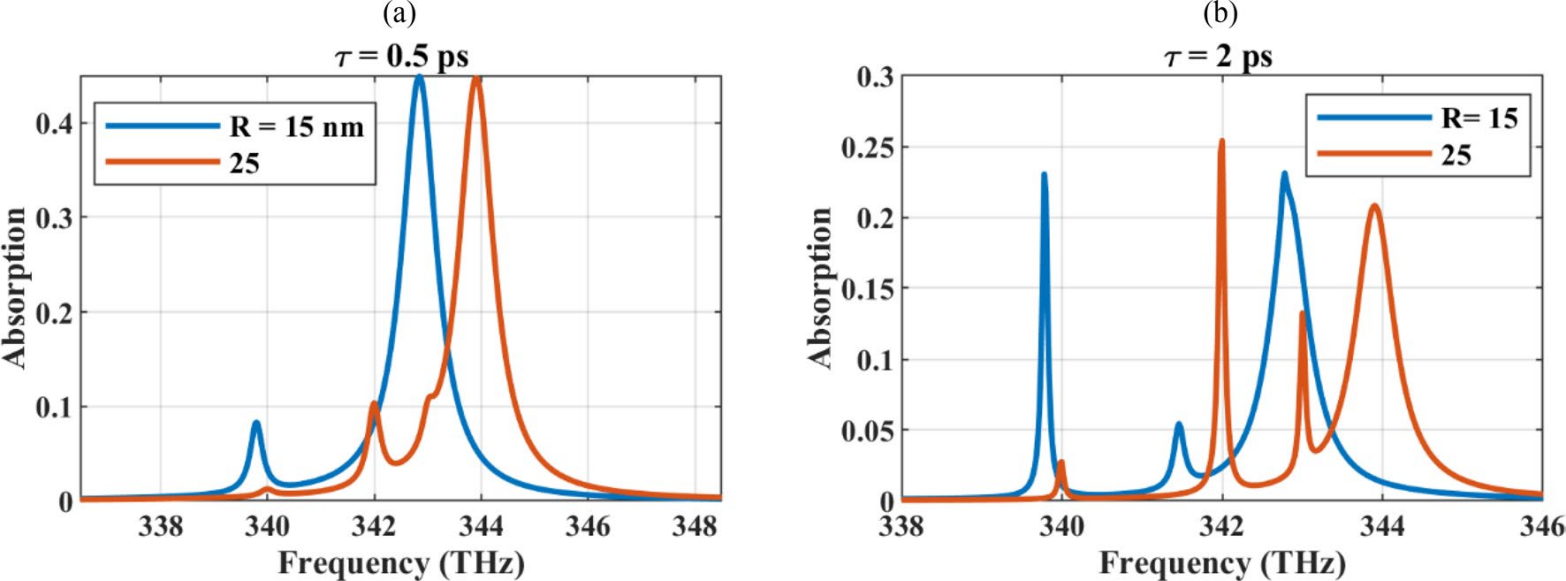
The absorption rate of the dense array with an interelement spacing of 5 nm studied for different core radii (**a**) *τ* = 0.5 ps and (**b**) *τ* = 2 ps.

The reported absorption spectrum is single-peak in which the cooperative and near-field effects are not visible. To understand this behavior, the absorption rate of the dense array with an interelement spacing of 5 nm was studied for different core radii in Fig. 8a,b, respectively for *τ* = 0.5 ps and *τ* = 2 ps. As the figure confirms, larger particles, exhibit multiple absorption peaks, as a result of near-field effects. The blue shift of the resonance spectrum by increasing the particle size is again confirmed for the array arrangement. The single peak behavior is also reported for the micron-sized plasmonic wires, where, the symmetry breaking has resulted in the resonance splitting^86^. Also, multiple invisibility regions are induced by symmetry breaking in a trimer of subwavelength graphene-coated nanowires^87^.

**Figure 9:**
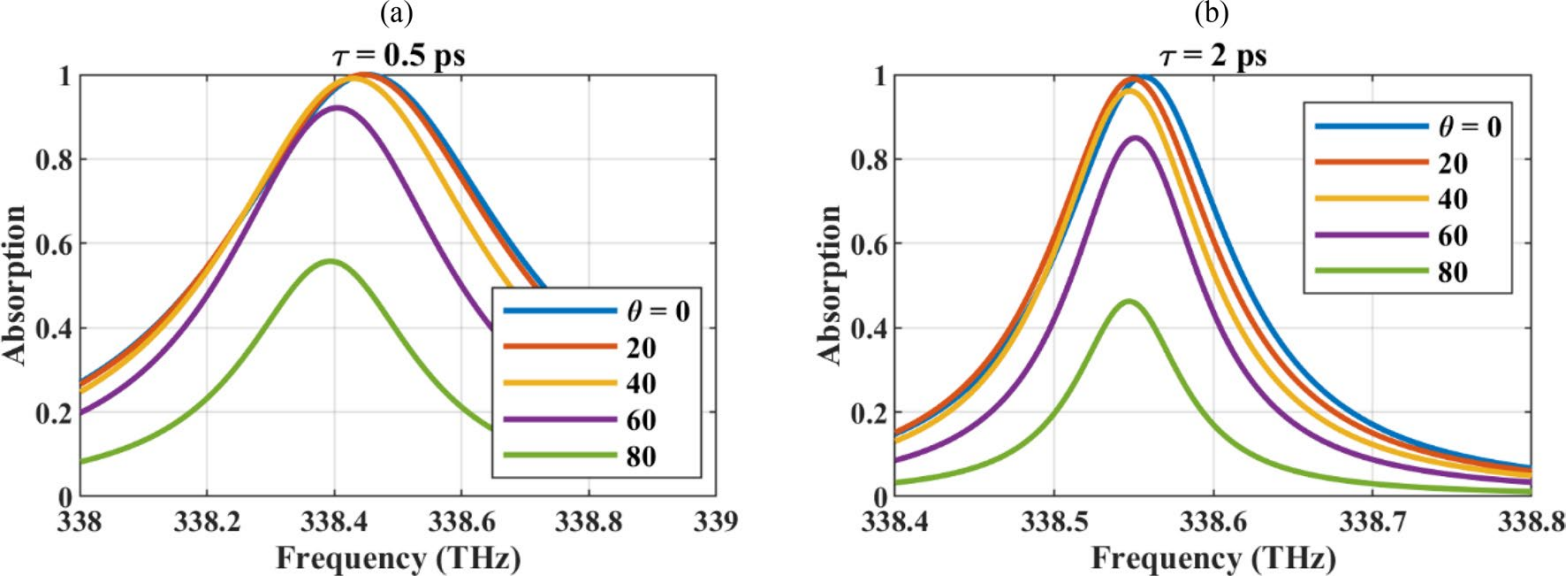
The perfect absorption achieved by residing the dense array on top of the substrate and its sensitivity to the incident angle (**a**) *τ* = 0.5 ps and *τ* = 2 ps.

To reach a perfect absorber, the nano-wires resided on top of a metal-backed dielectric substrate, with *ε* = 2 and the optimized height of *h* = 70 nm and 30 nm respectively for *τ* = 0.5 ps and 2 ps, are used. The perfect absorption rate of the device for dense arrays with interelement spacing of 5 nm is illustrated in Fig. 9a,b. The sensitivity of the absorption rate to the incident angle of the incoming wave is also shown in this figure, confirming above 90% absorption rate up to the angles around 60° and 50° respectively for *τ* = 0.5 and 2 ps. Perfect absorption can also be achieved by the sparse array and for other periodicities.

## Conclusion

Epsilon-near-zeros cylindrical nano-shells offer the opportunity for plasmonic absorption enhancement with reconfigurable characteristics at the near-infrared and visible spectrum. The resonance frequency of the excited plasmons blue-shifts as the particle radius increases, resulting in reaching a higher absorption efficiency with smaller particles. This exotic feature is achieved due to the utilization of higher-order modes for the absorber design. The operating spectrum can be modulated by graphene optical parameters. Moreover, perfect absorbers can be obtained by arranging the particles in a one-dimensional array on top of the metal-backed substrate. As the array becomes denser, spectral broadening and redshift of resonance frequency are observed. Considering the sparse array, the absorption spectrum is narrow and it requires high-quality graphene material to reach a higher absorption rate. Conversely, in the dense array, interelement mutual coupling results in spectral broadening and demands adjusting the graphene quality for higher enhancement. The use of small-sized particles in the absorber design results in a clear single peak in which the multiple scattering effect is not visible.

## Data Availability

The data associated with the present paper is available upon request from the corresponding author.
